# HIV-1 Genetic Diversity and Transmitted Resistance to Integrase Strand Transfer Inhibitors in Benguela, Angola

**DOI:** 10.3390/microorganisms14051156

**Published:** 2026-05-20

**Authors:** Isabel S. Godinho, Gonçalo Queirós, Lesya Yefimenko, Filomena M. Pereira, João Piedade

**Affiliations:** 1 Instituto de Higiene e Medicina Tropical, IHMT, Universidade NOVA de Lisboa, UNL, Rua da Junqueira 100, 1349-008 Lisboa, Portugal; mariasargodinho@gmail.com (I.S.G.); a596327@gmail.com (L.Y.); 2 Global Health and Tropical Medicine, GHTM, Associated Laboratory in Translation and Innovation Towards Global Health, LA-REAL, Instituto de Higiene e Medicina Tropical, IHMT, Universidade NOVA de Lisboa, UNL, Rua da Junqueira 100, 1349-008 Lisboa, Portugal; goncalopinhoqueiros@gmail.com (G.Q.); flmpereira@ihmt.unl.pt (F.M.P.)

**Keywords:** HIV-1, Angola, integrase, genetic diversity, integrase strand transfer inhibitors, drug resistance mutations

## Abstract

Angola is one of the countries with the highest HIV-1 genetic diversity, yet the implications of this diversity for antiretroviral therapy remain insufficiently characterised. Following the introduction of dolutegravir (DTG) in Angola in 2021, evaluating transmitted drug resistance prior to its widespread implementation is essential to inform treatment strategies and establish a baseline for future surveillance. In this study, 243 blood samples were collected from treatment-naïve people living with HIV attending the General Hospital of Benguela, Angola. The integrase coding region of proviral DNA was amplified and sequenced using the Sanger method. Phylogenetic relationships were inferred using a maximum likelihood approach, recombinant forms were characterised by bootscanning analysis, and resistance-associated mutations to integrase strand transfer inhibitors were identified using Stanford HIVdb, ANRS-MIE, and IAS-USA algorithms. A total of 92 integrase sequences were successfully obtained, revealing 16 distinct genetic forms, with unique recombinant forms accounting for 50.0%, followed by subtype C (10.9%) and sub-subtype F1 (8.7%). Five accessory mutations (L74I, L74M, Q95K, T97A, and E157Q) and one major mutation (E92G) were detected, corresponding to an overall prevalence of 28.8% (23/80). These findings highlight the extensive HIV-1 genetic complexity in Angola and support the continued use of DTG-based regimens, while underscoring the importance of sustained surveillance of integrase inhibitor resistance.

## 1. Introduction

HIV-1 (human immunodeficiency virus type 1) originated from multiple zoonotic transmissions of simian immunodeficiency virus (SIV) from non-human primates to humans in Central Africa. These events led to the emergence of distinct genetic groups—M, N, O, and P—of which group M became the globally dominant pandemic lineage [1]. As a result, Central Africa harbours the highest HIV-1 genetic diversity worldwide, with the circulation of nearly all recognised subtypes [2]. Across the African continent, subtype distribution varies considerably: Southern Africa is almost exclusively dominated by subtype C, subtype A predominates in Eastern Africa, and CRF02_AG is widespread in West and Central Africa. In Northern Africa, subtype C has increased in prevalence in recent years, replacing subtype B as the most frequently detected subtype [3]. Additionally, the proportion of recombinant viruses has increased substantially across Africa, reflecting a trend also observed on a global scale [4].

Since 2010, the number of annual HIV-1 infections has declined by approximately 40%. Nevertheless, sub-Saharan Africa remains the most affected region globally, with an estimated 25.3 million people living with HIV-1 and 650,000 new infections reported in 2024 [5]. In Angola, the epidemic is generalised, with an estimated 370,000 people living with HIV in 2024, and a relatively stable prevalence of 1.6% among adults aged 15–49. Recent estimates indicate approximately 21,000 new HIV infections annually, reflecting a decline compared to previous years, alongside an incidence of 0.6 per 1000 population. Women remain disproportionately affected, accounting for a higher share of infections. Although detailed national data on transmission routes are limited, heterosexual transmission is considered the predominant mode. Despite progress in reducing new infections and AIDS-related deaths, which were estimated at 13,000 in 2024, significant challenges persist in prevention, testing, and treatment coverage [6]. The country’s HIV-1 epidemic is characterised by extensive genetic diversity, including a predominance of subtype C, subtype A, and sub-subtype F1, alongside multiple unique recombinant forms (URFs) [7]. However, this subtype distribution is heterogeneous across the country: northern and central regions exhibit higher genetic diversity, whereas the southern region is largely characterised by a subtype C-dominated epidemic [8].

HIV-1 integrase (IN), a viral enzyme responsible for the integration of viral DNA into the host cellular genome, represents an optimal target for antiretroviral therapy due to the absence of a human homologue [9]. Integrase strand transfer inhibitors (INSTIs) block this process, thereby interrupting the viral replication cycle. Since the approval of the first INSTI, raltegravir (RAL), in 2007, several additional inhibitors have been introduced, including elvitegravir (EVG), dolutegravir (DTG), bictegravir (BIC), and cabotegravir (CAB) [10]. These drugs are commonly classified into first-generation (RAL and EVG) and second-generation (DTG, BIC, and CAB) INSTIs. While INSTIs as a drug class are characterised by favourable tolerability profiles and potent virological suppression, second-generation agents exhibit a higher genetic barrier to resistance and improved resistance profiles compared with first-generation compounds. Following WHO recommendations for the transition to DTG-based regimens as preferred first-line therapy, many sub-Saharan African countries, including Angola, progressively adopted DTG into their national treatment guidelines. In Angola, DTG-based regimens were formally introduced in January 2021 as part of the national antiretroviral programme, representing the first and, to date, only INSTI incorporated into routine clinical use in the country [11,12]. This transition aligned with global efforts to improve treatment outcomes, simplify regimens, and address rising levels of resistance to non-nucleoside reverse transcriptase inhibitors (NNRTIs).

Despite the high potency of INSTIs, HIV-1 can develop drug resistance mutations (DRMs) to this drug class. Such mutations may arise under conditions of incomplete viral suppression, leading to acquired drug resistance (ADR), or may be present at the time of infection when an individual acquires a virus already harbouring DRMs, resulting in transmitted drug resistance (TDR). DRMs are generally categorised as major mutations, which directly reduce drug susceptibility, and minor or accessory mutations, which compensate for fitness costs and may facilitate resistance pathways, potentially contributing to virological failure. Key INSTI-associated resistance mutations are curated by international expert panels and interpretation algorithms, including ANRS-MIE (*Agence Nationale de Recherche sur le SIDA et les Hépatites Virales-Maladies Infectieuses Emergentes*, France) [https://hivfrenchresistance.org/hiv-french-resitance-tables-of-rules/ (accessed on 15 November 2025)], IAS-USA (International Antiviral Society, USA) [13], and Stanford University HIV Drug Resistance Database (HIVdb) [14], which play a central role in the surveillance and interpretation of DRM patterns.

Pre-treatment screening for TDR is particularly important to prevent virological failure and onward transmission. However, its implementation remains limited in many low- and middle-income settings due to financial and technical constraints. The vulnerability of HIV treatment and prevention programmes to funding disruptions became especially evident in January 2025, when the U.S. President’s Emergency Plan for AIDS Relief (PEPFAR) experienced a temporary suspension [15], with immediate repercussions for HIV-related services in several settings [5]. Angola is among the 20 countries with the highest dependence on United States Agency for International Development (USAID) funding, ranking ninth, with 34% of HIV programme resources originating from U.S. support [16]. This context underscores the urgency of strengthening locally generated evidence on HIV prevention, including data on the prevalence of TDR.

In this cross-sectional study, conducted at the General Hospital of Benguela, in the city of Benguela (Angola) and the Institute of Hygiene and Tropical Medicine, NOVA University Lisbon (Portugal), blood samples were collected from treatment-naïve people living with HIV (PLWH). The study aimed to (i) update local HIV-1 subtype diversity, including the characterisation of potential recombinant forms, and (ii) investigate the presence of TDR to INSTIs. Given the delayed introduction of this drug class in Angola, the findings are intended to address existing knowledge gaps and support informed treatment strategies for HIV-1 in this region.

## 2. Materials and Methods

### 2.1. Study Population, Sample Collection and Screening

This study was conducted within a larger cross-sectional project approved by the Scientific and Ethics Committee of the General Hospital of Benguela. A total of 243 blood samples were collected from individuals newly diagnosed with HIV-1 infection who had no prior history of antiretroviral therapy, recruited from inpatient wards, outpatient clinics, and the emergency department. Peripheral blood was obtained by venipuncture between August 2016 and January 2017. All participants or their legal guardians provided written informed consent prior to enrolment and completed a structured questionnaire to collect general sociodemographic and epidemiological data.

Initial screening for HIV-1/2 antibodies was performed using two third-generation rapid immunochromatographic assays: VIH 1/2 (Healgen Scientific, Houston, TX, USA) and Anti-HIV 1/2 Test (Türklab, Menderes, Türkiye). Samples reactive in at least one assay were further processed. Whole blood was spotted onto QIAcard^™^ FTA^™^ Elute Micro Cards (indicating) (Qiagen, Hilden, Germany) for nucleic acid preservation at room temperature. In parallel, serum from positive samples was separated by centrifugation and stored at −20 °C until further analysis. For confirmatory testing and external validation, a third rapid assay—Hexagon HIV-1/2 (Human Diagnostics, Wiesbaden, Germany)—was performed at the Institute of Hygiene and Tropical Medicine, NOVA University Lisbon, Lisbon, Portugal.

### 2.2. FTA^™^ Card Processing

FTA^™^ cards were processed according to the manufacturer’s instructions, with minor modifications. Briefly, punched FTA^™^ card discs were washed using QIAcard^™^ FTA^™^ Wash Buffer (Qiagen, Hilden, Germany) and subsequently used directly as a template for PCR amplification.

### 2.3. Nested PCR Amplification and Sequencing of the HIV-1 Integrase Region

A nested PCR protocol was set up to amplify the HIV-1 integrase (IN) coding region from proviral DNA purified from dried blood spots (FTA^™^ cards). Amplification reactions were performed using Supreme NZYTaq II 2× Green Master Mix (NZYtech, Lisbon, Portugal). Primer sequences (Table 1) were obtained from the ANRS-MIE HIV drug resistance research consortium. Following optimisation of annealing temperatures and primer combinations, the final nested PCR conditions were established. The first-round reaction used primers INPS1 and INPR8, whereas the second-round reaction used INPS3 and INPR9. Annealing temperatures were set at 50 °C for the first round and 55 °C for the second, yielding a 723 bp amplicon. Thermal cycling consisted of an initial denaturation at 95 °C for 5 min, followed by 35 cycles of denaturation at 94 °C for 30 s, annealing at the respective temperatures for 30 s, and extension at 72 °C for 30 s, with a final extension step at 72 °C for 7.5 min. Negative controls were included in each run to monitor potential contamination.

Amplicons of the expected size were confirmed by electrophoresis on a 1.5% (*w*/*v*) agarose gel and subsequently purified and subjected to bidirectional Sanger sequencing at STAB VIDA (Caparica, Portugal) [17].

### 2.4. Sequence Editing and Analysis

Raw sequence chromatograms obtained from bidirectional sequencing were assembled and edited using BioEdit Sequence Alignment Editor v.7.2.5 [18], and consensus sequences were generated for each sample. Sequence identity was subsequently verified using the Basic Local Alignment Search Tool (BLASTn) v.2.16.0 (NCBI, Bethesda, MD, USA), to confirm alignment with HIV-1 integrase (pol) sequences [19]. Sequence identifiers were formatted using the prefix “AO”, representing the ISO 3166-1 [20] alpha-2 country code for Angola, followed by the corresponding sample number.

Multiple sequence alignments were generated using MAFFT v.7 [21], incorporating both the study-derived sequences and a curated set of reference sequences representing well-characterised pure subtypes and circulating recombinant forms (CRFs). These reference sequences were retrieved from GenBank [22] and the Los Alamos HIV Sequence Database (https://www.hiv.lanl.gov/, accessed on 30 May 2025), selected based on phylogenetic proximity and geographical relevance to the study sequences. To refine the alignment, poorly aligned or highly divergent regions were excluded using Gblocks v.0.91b [23].

Phylogenetic trees were then inferred using the maximum likelihood (ML) method implemented in IQ-TREE [24]. Model selection was performed using the Bayesian Information Criterion (BIC) implemented in IQ-TREE. Branch support was assessed by bootstrap analysis with 1000 replicates. The optimal nucleotide substitution model, as determined by IQ-TREE, was the General Time Reversible (GTR) model, which accounts for different substitution rates and unequal base frequencies. Phylogenetic trees were visualised and edited using FigTree v.1.4.4 (http://tree.bio.ed.ac.uk/software/figtree/, accessed on 30 May 2025). Where necessary, recombination analysis was performed using SimPlot v.3.5.1 [25], applying the neighbour-joining method with 1000 bootstrap replicates, a sliding window of 100–200 nucleotides, and step sizes of 10–20 nucleotides.

The HIV-1 sequences generated in this study have been deposited in GenBank under accession numbers PX681887 to PX681970 (Table S1).

### 2.5. Detection of INSTI Resistance-Associated Mutations

Putative amino acid sequences were inferred from nucleotide sequences by translation and analysed for mutations associated with reduced susceptibility to INSTIs, as well as for other genetic polymorphisms and mutations, including APOBEC-induced hypermutation [26]. This analysis was performed using the HIVdb Genotypic Resistance Interpretation Algorithm from the Stanford University HIV Drug Resistance Database v.10.1 [14]. This tool was used to identify major and accessory drug resistance mutations (DRMs), as well as other mutations not directly associated with antiretroviral (ARV) resistance. The algorithm assigns a score to each mutation for specific INSTIs, allowing estimation of the corresponding phenotypic resistance level for each sequence under analysis. Based on this scoring system, resistance was classified as susceptible, potential low-level resistance, low-level resistance, intermediate resistance, or high-level resistance. Sequences were also screened for APOBEC-mediated hypermutation [26] using the same algorithm and those exhibiting such patterns, as well as sequences containing stop codons not directly dependent on APOBEC activity, and indicative of defective viral genomes, were excluded from the resistance mutation analysis.

Considerations regarding DRMs were additionally informed by reference to mutation lists from ANRS-MIE [https://hivfrenchresistance.org/hiv-french-resitance-tables-of-rules/ (accessed on 15 November 2025)] and IAS-USA [13] to support interpretation of the identified mutations.

### 2.6. Statistical Analysis

To characterise the study population, distributions of demographic and epidemiological variables were analysed, including categorical variables such as sex, age, marital status, educational level, and presence of other diagnosed sexually transmitted infections. For age, in addition to distribution analysis, the mean and standard deviation were calculated.

## 3. Results

### 3.1. Sociodemographic and Epidemiological Analysis

This study included blood samples collected from 243 individuals newly diagnosed with HIV-1 infection, attending the General Hospital of Benguela, in Angola. All participants were Angolan, ranging in age from 0 to 95 years (mean age: 38 ± 19.7 years); 39.1% were male and 53.9% female (7.0% unspecified). All participants resided in the province of Benguela, with 61% living in urban areas and 41% in the city of Benguela, the provincial capital. As part of a broader cross-sectional study on sexually transmitted infections, data on co-infection with syphilis and hepatitis B were also collected. Among the 226 participants tested for these infections, co-infection was observed in 17% (39/226). Of these, 54% (21/39) had syphilis, 41% (16/39) had hepatitis B, and 5% (2/39) were co-infected with HIV-1, syphilis, and hepatitis B. Core data were compiled in Table 2.

### 3.2. Phylogenetic Characterisation of the Sequences

From the 243 FTA^™^ card discs processed, 92 high-quality sequences were obtained, representing approximately 38% of the total sample set. Sequence lengths ranged from 642 to 685 bp.

**Figure 1:**
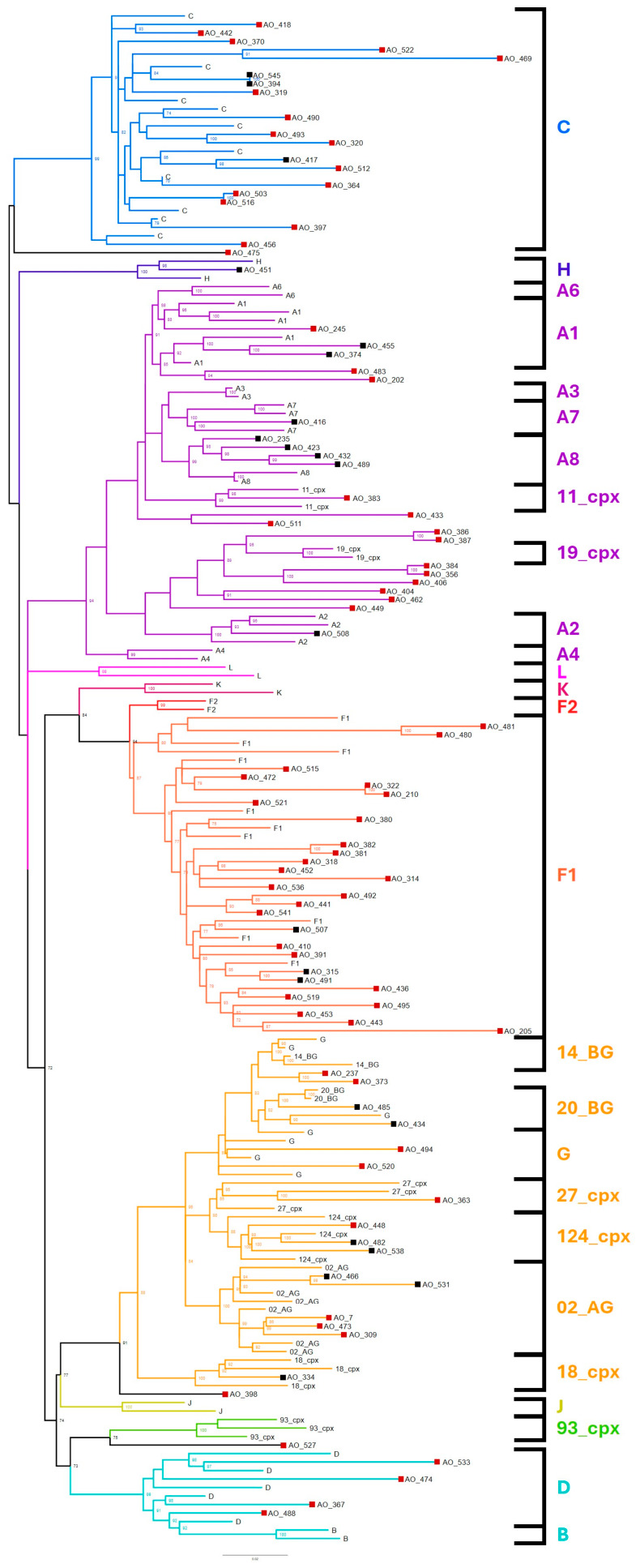
Maximum likelihood (ML) phylogenetic tree inferred from 181 HIV-1 integrase sequences, including 92 study sequences and 89 reference sequences. Subtype lineages are distinguished by colour. Study sequences classified with strong bootstrap support are indicated by black squares (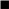), whereas unclassified sequences selected for bootscan analysis are indicated by red squares (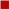). The tree was midpoint-rooted and, for clarity, bootstrap values < 70% were not displayed.

To perform phylogenetic analyses, reference HIV-1 sequences representing subtypes A-L and major CRFs were retrieved from the Los Alamos HIV Sequence Database. To ensure adequate representation of sequences with unusual or divergent characteristics, additional reference sequences were obtained from GenBank, guided by BLAST similarity searches. This process yielded 89 reference sequences, which, together with the 92 study-derived sequences, resulted in a dataset of 181 sequences. After alignment and trimming, a final 634 bp-long dataset was obtained, corresponding to positions 4418–5059 of the HXB2 reference genome with the exclusion of eight hypervariable sites. This dataset was used to infer the maximum likelihood phylogenetic tree shown in Figure 1.

Most subtype reference sequences formed well-supported monophyletic clusters. Exceptions were observed for sub-subtype A1, which separated into two distinct clades, and for subtypes B and D, which clustered together, consistent with their close genetic relationship. The clustering pattern of CRFs generally reflected their mosaic genomic composition for the integrase region. Specifically, CRFs 02_AG, 14_BG, 18_cpx, 20_BG, 27_cpx, and 124_cpx grouped with subtype G, consistent with the predominance of G-derived sequences in the region studied, whereas CRFs 11_cpx and 19_cpx clustered with subtype A. CRF93_cpx grouped with subtypes D/B, a pattern likely influenced by the phylogenetic position of sequence AO527, which is further characterised below as a recombinant involving CRF19_cpx and subtype D. A total of 22 sequences (22/92; 23.9%) were directly assigned a subtype or CRF based on phylogenetic tree interpretation. These comprised subtypes A1 (2/22; 9.1%), A2 (1/22; 4.5%), A7 (1/22; 4.5%), A8 (4/22; 18.2%), C (3/22; 13.6%), F1 (3/22; 13.6%), G (1/22; 4.5%), and H (1/22; 4.5%), as well as CRFs 02_AG (2/22; 9.1%), 18_cpx (1/22; 4.5%), 20_BG (1/22; 4.5%), and 124_cpx (2/22; 9.1%).

**Figure 2:**
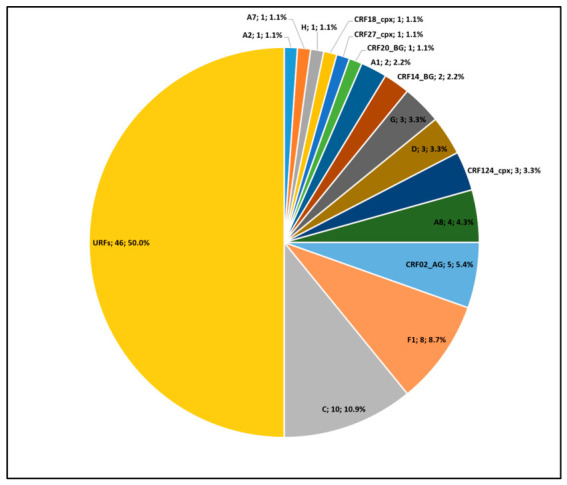
Distribution of HIV-1 subtypes, sub-subtypes and recombinant forms based on phylogenetic analysis complemented by SimPlot recombination profiling.

To investigate potential recombination events and establish accurate subtype classification, the 70 unclassified sequences (70/92; 76.1%) highlighted in the phylogenetic tree (Figure 1) were subjected to bootscanning analysis using the same reference panel employed for phylogenetic reconstruction. Genomic regions lacking consistent subtype assignment were designated as undetermined (U). Recombination analysis revealed that the majority of previously unclassified sequences corresponded to recombinant forms (Figure S1). Exceptions included AO7, AO309, AO473, and AO531, which are most consistent with CRF02_AG; AO237 and AO373, which are compatible with CRF14_BG or a subtype G lineage related to this CRF; AO314, AO391, AO410, AO452, and AO541, classified as sub-subtype F1; AO320, AO370, AO418, AO442, AO490, AO493, and AO512, assigned to subtype C; AO363, classified as CRF27_cpx; AO367 and AO533, classified as subtype D; AO448, which was most closely related to CRF124_cpx or to a CRF27_cpx-related lineage; and AO494 and AO520, assigned to subtype G. Finally, sequences AO433 and AO511, which appeared to share a similar recombination pattern, were conservatively classified as U due to their high mosaic complexity. These results were used to update subtype proportions, as summarised in Figure 2, with individual sequence classifications compiled in Table S1.

### 3.3. Resistance Analysis to INSTIs

A bioinformatic analysis was performed on 92 translated putative amino acid sequences to identify mutations associated with resistance to INSTIs. Most sequences (90/92; 97.8%) corresponded to a 228-residue fragment of the 288 amino acids comprising the full-length integrase (IN) enzyme, representing approximately 79% of its primary structure. Despite the partial coverage of IN (positions 54–281), this fragment encompasses nearly all positions at which major and accessory drug resistance mutations (DRMs) have been described. Nevertheless, among the 60 amino acid positions not covered are codons 49 and 51, which have been reported to harbour the accessory DRMs A49G and H51Y, both of which are extremely rare non-polymorphic variants [27]. Twelve sequences (12/92; 13.0%) were excluded from the resistance analysis, eleven due to evidence of APOBEC-mediated hypermutation [26], identified as excessive G-to-A substitution patterns, and one due to the presence of an unusual stop codon. Using the same algorithm, predicted phenotypic resistance profiles for each INSTI were generated for all analysed sequences.

Among the 80 putative IN amino acid sequences analysed, six distinct mutations associated with reduced susceptibility to INSTIs were identified in 23 sequences (Table 3). These comprised one major DRM, E92G (1/80; 1.3%), and five minor DRMs: L74I (14/80; 17.5%), L74M (3/80; 3.8%), Q95K (2/80; 2.5%), T97A (3/80; 3.8%), and E157Q (1/80; 1.3%). Each mutation was detected individually, with no co-occurrence of DRMs observed in any sequence, with the exception of AO473, which harboured L74I and T97A.

**Figure 3:**
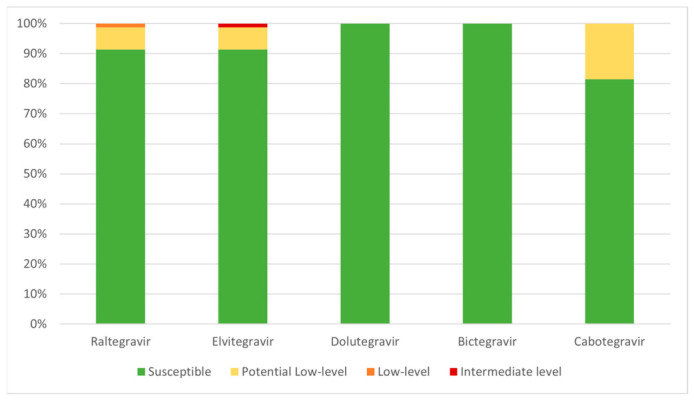
Distribution of predicted phenotypic resistance levels for each INSTI based on HIVdb interpretation.

Using the Stanford HIV Drug Resistance Database (HIVdb) algorithm, resistance scores were assigned to each identified DRM for individual INSTIs, allowing prediction of phenotypic resistance levels (Figure 3). Most viruses (60/80; 75.0%) were predicted to be fully susceptible to all INSTIs analysed, whereas 20/80 (25.0%) were predicted to show some degree of phenotypic resistance. The three sequences listed in Table 3 harbouring the L74M mutation were classified as susceptible, as the resistance scores assigned to this mutation by the Stanford HIVdb algorithm do not confer any level of reduced susceptibility when present alone.

The major mutation E92G conferred intermediate resistance to elvitegravir (EVG) (score 30), low-level resistance to raltegravir (RAL) (score 15), and potential low-level resistance to cabotegravir (CAB) (score 10). The accessory mutation L74I was associated with potential low-level resistance to CAB (score 10), while L74M was assigned a score of 5 for all INSTIs, which, per se, does not correspond to a defined resistance category. Mutations Q95K, T97A, and E157Q were each associated with potential low-level resistance to EVG (score 10) and RAL (score 10). Predicted phenotypic resistance levels for each INSTI and their proportions (Figure 3) were determined according to the scoring system described. Overall, transmitted drug resistance (TDR) to RAL and EVG was observed in 8.8% (7/80) of sequences, while TDR to CAB was detected in 18.8% (15/80). No TDR was identified for DTG or BIC.

In addition to these DRMs, two polymorphisms were identified (Table 3). The S119R substitution is described as weakly selected by INSTIs, with an arguable impact on susceptibility in combination with other mutations [28]. S230N is likewise not associated with INSTI resistance [29]. No additional mutations listed by ANRS-MIE or IAS-USA were identified.

## 4. Discussion

This study represents the first characterisation of transmitted drug resistance (TDR) to INSTIs prior to their introduction in Angola, while also providing a comprehensive analysis of the circulating HIV-1 subtypes and recombinant forms in the region. The study was based on 243 blood samples from the province of Benguela, Angola, of which 92 yielded high-quality IN-encoding sequences. Although the amplification rate was suboptimal (92/243; 38%), this is not unexpected, as multiple factors can hinder proviral DNA amplification. These include the age of the samples, which were collected between late 2016 and early 2017, potentially low viral loads, which were not measured at the time of collection, and the extensive intrinsic genetic diversity of HIV-1. Many laboratory protocols and phylogenetic tools were originally optimised for subtype B viruses, which are uncommon in Central Africa, potentially introducing methodological bias.

The initial phylogenetic analysis revealed a very high diversity of HIV-1 subtypes. The initial reference dataset used for maximum likelihood (ML) tree construction included all circulating recombinant forms (CRFs) reported to originate or circulate in sub-Saharan Africa, as well as CRFs described in countries with historical and epidemiological links to Angola, such as Portugal, Brazil, and Cuba. CRFs that did not provide relevant phylogenetic information were removed to improve clarity. Overall, the phylogenetic tree (Figure 1) exhibited well-supported topology, with most subtypes and CRFs forming monophyletic clusters with strong bootstrap values (>70%). An exception was sub-subtype A1, which formed a paraphyletic group within a larger monophyletic cluster that included A6. This pattern has been reported previously and likely reflects the close phylogenetic relationship between A1 and A6. In fact, there is strong evidence that the A6 sub-subtype originated from an African A1 lineage and subsequently became prevalent in countries of the former Soviet Union, which historically maintained significant political and social ties with several African nations [30].

Twenty-two sample sequences belonging to subtypes A, C, F, G, and H were identified based on tree topology and bootstrap support, with additional sequences potentially corresponding to CRF02_AG, CRF18_cpx, CRF20_BG, and CRF124_cpx (Figure 1). Other sequences segregated within supported clades but could not be confidently assigned to a subtype or CRF due to factors such as branch length or lack of close, well-supported ancestral nodes, indicating a significant degree of genetic divergence. These unclassified sequences were subsequently analysed for APOBEC-induced mutations and recombination, both of which could contribute to the observed genetic distances.

Eleven sequences contained at least one mutation flagged by the HIVdb algorithm as APOBEC-mediated: AO205, AO356, AO363, AO441, AO443, AO469, AO474, AO482, AO491, AO522, and AO533. Among these, three sequences—AO205, AO469 and AO522—harboured 15 or more mutations, reflected in the particularly long branch length in the phylogenetic tree (Figure 1). These sequences were excluded from the resistance analysis, as APOBEC-mediated damage [26] can introduce mutations that are biologically irrelevant, likely rendering the virus replication-incompetent.

After bootscanning analysis of the remaining 70 sequences, 24 were assigned to a pure subtype or a previously described CRF: seven to subtype C, two to subtype D, five to sub-subtype F1, two to subtype G, four to CRF02_AG, two to CRF14_BG, one to CRF27_cpx, and one to CRF124_cpx. The remaining sequences displayed recombination patterns and were therefore considered unique recombinant forms (URFs). Overall, nine pure subtypes were identified, namely A1, A2, A7, A8, C, D, G, H, and F1. A considerable number of CRFs were also observed, though caution is required when interpreting the classification of such CRFs, specifically of CRF14_BG, CRF20_BG, and CRF124_cpx. The fragment used for the phylogenetic analysis spans most sites between nucleotides 4418 and 5059 (in HIV-1 HXB2), which does not include a recombination breakpoint for the CRFs mentioned above. Consequently, these sequences might instead belong to the parental pure subtype that gave rise to the recombinants. CRF14_BG was originally characterised using sequences from Spain [31] and is currently circulating in Europe, particularly in the Iberian Peninsula. Evolutionary analyses suggest that its parental G strain likely originated from Angola during the post-colonial exodus following independence [32], supporting the idea that these sequences may represent a G subtype lineage related to this parental strain rather than the CRF itself. Similarly, CRF20_BG, described in Cuban sequences [33], is mainly observed in the Caribbean. However, its closest G subtype relatives are Iberian, which, combined with the historical Angolan origin of the G lineage that gave rise to CRF14_BG [32], suggests that the parental G strain of CRF20_BG may also have come from Angola during the significant migration of Cubans in the 1980s. This interpretation is supported by the ML tree (Figure 1), where both CRF14_BG and CRF20_BG form a strongly supported monophyletic group together with G sequences from Portugal, Spain, and Cuba, indicating that these sequences may not represent true CRF20_BG. Finally, CRF124_cpx was first described in Angola [34] and remains largely restricted to the country. Its parental lineages likely originated in the Democratic Republic of Congo (DRC), including the integrase region, which is entirely classified as CRF27_cpx. Given that both CRF124_cpx and CRF27_cpx co-circulate in Angola and DRC, raising doubts on the location of the original recombination event, it remains uncertain whether sequences clustering with CRF124_cpx truly represent this second-generation recombinant or rather CRF27_cpx lineages related to its formation.

URF patterns identified by bootscanning exhibited substantial subtype diversity, with some mosaics incorporating previously described CRFs (Figure S1). This degree of complexity was expected, as previous molecular epidemiology studies reported similarly diverse URF profiles in the region [7]. In the recombination patterns described here, subtype F1 (20/46; 43.5%), C (15/46; 32.6%), and G (14/46; 30.4%) were the most commonly found, consistent with their known regional prevalence [7,8]. Several URFs, particularly those sharing strongly supported nodes and shorter branch lengths, displayed identical mosaic patterns that were consistent with their phylogenetic placement within subtype clades. For example, AO527 segregated prematurely in the CRF93_cpx cluster (bootstrap 75%) and exhibited a mosaic comprising subtypes D, A8, and CRF93_cpx segments. This may explain the proximity of the CRF93_cpx cluster to subtype D in the phylogenetic tree (Figure 1). Similarly, AO245 presented an A6/A1 mosaic pattern and was positioned between the A6 cluster and one of the A1 clusters with strong bootstrap support. Although this may represent recombination between A sub-subtypes, it could also reflect the evolutionary divergence of A1 into A6, as previously discussed [30]. Further temporal and phylogeographical analyses would be required to distinguish between both possibilities.

Some URF groups sharing identical recombination patterns (Figure S1) may indicate the emergence of new CRFs in the region. The criteria for defining a new CRF include three epidemiologically unlinked near full-length genome sequences [35]. In the present study, two sets of sequences met the numerical criterion: AO356, AO384, and AO406 (CRF19_cpx/G mosaic), and AO436, AO443, AO453, AO495, and AO519 (F1/C/F1 mosaic). Both clusters formed well-supported monophyletic groups (bootstrap 100% and 93%, respectively). Confirmation of their status as novel CRFs would require assessment of epidemiological independence and full-length genome sequencing. Additional pairs of sequences shared recombination patterns (Figure S1) but did not meet the minimum numerical requirement for CRF designation. These include AO210/AO322 (F1/G), AO381/AO382 (F1/A8/F1), AO386/AO387 (A2/G/A2), AO404/AO462 (A2/G/A2), AO436/AO516 (F1/C/F1), AO441/AO492 (F1/CRF93_cpx/F1), AO480/AO481 (F1/G/F1), and AO503/AO516 (C/L/C). One additional pair, AO469/AO522, was excluded from this listing due to lower confidence in bootscanning results, likely influenced by extensive APOBEC-mediated hyperadenylation reflected in their long branch lengths. Notably, the pair AO386/AO387 shares a strongly supported node (bootstrap 95%) with the CRF19_cpx cluster. Although the supporting branch is relatively long and CRF19_cpx was originally described in Cuba [36], the similarity between this mosaic structure and the integrase region of the CRF19_cpx, which is also A/G/A, may suggest an ancestral relationship. Indeed, previous studies have proposed that the parental lineages contributing to CRF19_cpx originated in Central Africa [37], potentially involving an ancestral A/G recombinant.

Following phylogenetic analysis, the presence of INSTI-associated DRMs was assessed primarily using the HIVdb algorithm. Twenty-three sequences (23/80; 28.8%) harboured at least one DRM, of which 22 contained only accessory mutations and one carried a major DRM. The only major DRM detected was E92G, in sequence AO416. This is a surveillance DRM [27] which markedly reduces susceptibility to elvitegravir (EVG) while having a limited impact on susceptibility to other INSTIs [38].

L74I was the most frequently observed accessory mutation, identified in fourteen sequences (14/80; 17.5%). This mutation, with subtype-dependent prevalence, represents the consensus amino acid residue in sub-subtype A6, likely reflecting a founder effect during its expansion across countries of the former USSR [39]. In our dataset, L74I alone was associated with potential low-level resistance to cabotegravir (CAB), and in combination with mutations at positions 118, 148, or 155, may contribute to reduced susceptibility to all second-generation INSTIs [14]. Concordantly, the ANRS-MIE panel similarly recognises its role in resistance when in combination with other DRMs. The IAS-USA algorithm [13] classifies L74I as a signature mutation in sub-subtype A and considers it a major CAB-associated DRM only in this subtype, where it has been shown to restore replication capacity in CAB-resistant viruses carrying other DRMs [40]. Recent data further indicate that L74I is significantly associated with dolutegravir (DTG) resistance in African strains [41]. Collectively, its synergistic effects with other DRMs to reduce susceptibility to all INSTIs [42,43], documented role in sub-subtype A6 CAB-resistant viruses, and association with DTG resistance highlight the importance of monitoring this mutation, particularly given its significant prevalence in our study (17.5%).

L74M, an accessory mutation present in three sequences, was not initially associated with resistance [44]. However, its role as a compensatory mutation enhancing viral fitness in the presence of other DRMs has since been recognised and is reflected in current resistance scoring systems [45]. Q95K, another accessory mutation, detected in two sequences, is primarily selected by first-generation INSTIs raltegravir (RAL) and EVG. Although it does not substantially reduce susceptibility to these pharmaceuticals on its own, it contributes to resistance in combination with other mutations, partly through viral fitness compensation, with a more pronounced effect observed for EVG [46]. T97A, found in three sequences, has a modest impact on first-generation INSTIs when present alone [47], but exerts stronger synergistic effects in combination with other DRMs [45]. Finally, E157Q was identified in only one sequence. Similar to other accessory mutations, its independent effect on INSTI susceptibility is marginal. However, it has recognised potential to contribute to resistance when combined with additional DRMs [48].

Overall, our findings are consistent with the lower genetic barrier to resistance of first-generation INSTIs (RAL and EVG), which exhibited the same rate of transmitted drug resistance (TDR) (7/80; 8.8%), whereas no TDR was observed for DTG or bictegravir (BIC). The highest TDR proportion was observed for CAB (15/80; 18.8%). Although the overall TDR proportion was the same for RAL and EVG, the predicted level of resistance differed. Notably, one sequence showed intermediate-level resistance to EVG (Figure 3), a category associated with a high likelihood of reduced antiviral efficacy, although some residual activity may be retained. These findings are in line with other reports from sub-Saharan Africa describing a comparable prevalence and distribution of accessory INSTI-associated DRMs [49]. Importantly, these mutations appear to be naturally circulating in sub-Saharan Africa, including Angola, even prior to the widespread introduction of INSTIs as first-line therapy [50]. This is expected, as L74I, L74M, Q95K, T97A, and E157Q are predominantly characterised as accessory mutations with compensatory functions. As such, they exert minimal impact on viral fitness and are unlikely to adversely affect viral replicative capacity in the absence of selective pressure exerted by INSTIs. Furthermore, the implementation of DTG-based first-line regimens in Angola does not appear, to date, to have resulted in a measurable increase in reported DRMs or TDR [51].

Several limitations should be considered when interpreting our results. First, the use of proviral DNA as a template for molecular epidemiology analyses may lead to the valorisation of archived or replication-incompetent viruses, so DRM findings related to putative phenotypic resistance should be interpreted conservatively. Such viral variants are not necessarily of clinical significance, and archived viruses may carry resistance-associated mutations whose impact on long-term antiretroviral therapy remains uncertain. Second, the use of Sanger DNA sequencing has limited sensitivity and may underestimate minority viral populations, which often carry fitness-reducing DRMs. Moreover, as only a portion of the integrase-coding region was sequenced, subtype assignments are necessarily partial and should be considered provisional. Finally, although sociodemographic characteristics were included to provide context for the study population, no formal analysis was performed to assess their association with viral subtypes or the presence of DRMs. Exploring such relationships could offer valuable epidemiological insights and should be addressed in future studies with appropriate designs and statistical power.

## 5. Conclusions

This study provides important data on the molecular epidemiology of HIV-1 and transmitted drug resistance (TDR) to INSTIs in Angola, prior to the introduction of this drug class, and supports the continued use of dolutegravir (DTG) in first-line combination therapy. Most detected INSTI DRMs were accessory mutations—L74I (17.5%), L74M (3.8%), Q95K (2.5%), T97A (3.8%), and E157Q (1.3%)—with only one major DRM, E92G (1.3%). These mutations were primarily associated with potential low-level resistance to raltegravir (RAL), elvitegravir (EVG), and cabotegravir (CAB), with the exception of E92G, which conferred low-level resistance to RAL and intermediate-level resistance to EVG. Overall, no major resistance to second-generation INSTIs was observed. However, the notable prevalence of L74I warrants vigilance regarding potential resistance to DTG and CAB [40,41,42,43], underscoring the need for ongoing resistance surveillance. As this data precedes the widespread introduction of INSTIs in Angola, it provides a critical reference for monitoring future changes in resistance patterns.

Phylogenetic analysis revealed extensive HIV-1 genetic diversity, with the circulation of multiple pure subtypes (A1, A2, A7, A8, C, D, F1, G, and H), of which subtype C was the most prevalent (10.9%). A high level of recombinant diversity was also observed, including CRF02_AG, CRF18_cpx, CRF27_cpx, and CRF124_cpx, while unique recombinant forms (URFs) accounted for the largest proportion of sequences (50.0%). These findings of genetic diversity and recombinant complexity highlight the considerable HIV-1 variation in Angola, contributing to our understanding of regional and historical virus dissemination.

Together, the findings provide valuable baseline data on HIV-1 genetic diversity and INSTI TDR prior to changes in therapeutic guidelines in Angola, reinforcing the importance of ongoing molecular surveillance to guide effective treatment strategies in a highly diverse epidemic.

## Figures and Tables

**Table 1:** Description of the primers used in the nested PCR reaction.

Name	Sequence	Location (in HIV-1 HXB2)	IN Coding Region (in HIV-1 HXB2)
INPS1	5′-TAG TAG CCA GCT GTG ATA AAT GTC-3′	4336 → 4359	4230 → 5096
INPR8	5′-TTC CAT GTT CTA ATC CTC ATC CTG-3′	5082 → 5105	
INPS3	5′-GAA GCC ATG CAT GGA CAA G-3′	4371 → 4389	
INPR9	5′-ATC CTC ATC CTG TCT ACT TGC C-3′	5072 → 5093	

**Table 2:** Core demographic characteristics of the study population. Sex distribution is presented as counts and percentages, and age is categorised into predefined age groups to describe the population structure.

Characteristics	
*Sex*	*n* (*%*)
Male	95 (39.1)
Female	131 (53.9)
Unspecified	17 (7.0)
*Age* (*years*)	
0–5	13 (5.3)
6–14	21 (8.6)
15–24	30 (12.3)
25–34	36 (14.8)
35–49	43 (17.7)
≥50	79 (32.5)
Unspecified	21 (8.6)

**Table 3:** Drug resistance mutations (DRMs) and polymorphic mutations identified using the HIVdb algorithm. The major DRM is underlined.

Mutation		Sequence Number
DRMs	L74I	AO210, AO235, AO309, AO322, AO417, AO423, AO455, AO473, AO475, AO489, AO508, AO512, AO515, AO538
	L74M	AO391, AO434, AO472
	E92G	AO416
	Q95K	AO456, AO520
	T97A	AO448, AO473, AO480
	E157Q	AO404
Polymorphic	S119R	AO381, AO382, AO397, AO448, AO456, AO493
	S230N	AO381, AO382

## Data Availability

The HIV-1 integrase sequences presented in the study are openly available in GenBank under accession numbers PX681887 to PX681970, listed in Supplementary Materials (Table S1). The original contributions presented in this study are included in the article/Supplementary Materials. Further inquiries can be directed to the corresponding author.

## Supplementary Materials

The following supporting information can be downloaded at: https://www.mdpi.com/article/10.3390/microorganisms14051156/s1, Figure S1: SimPlot bootscanning profiles of sequences submitted to recombination analysis. The red line indicates the 70% bootstrap support threshold. Table S1: GenBank accession numbers and subtype/recombinant form classification of each sequence analysed.
