# Supplementary material for: HIV-1 Genetic Diversity and Transmitted Resistance to Integrase Strand Transfer Inhibitors in Benguela, Angola
Source: Microorganisms. 2026 May 20;14(5):1156. doi: 10.3390/microorganisms14051156 (PMC13209627; doi:10.3390/microorganisms14051156)
Supplement: Supplementary file 1 [file microorganisms-14-01156-s001.zip › microorganisms-4213716-supplementary.pdf]

microorganisms

MDPI

Supplementary Materials

HIV-1 Genetic Diversity and Transmitted Resistance to Integrase Strand Transfer Inhibitors in Benguela, Angola

Isabel S. Godinho [1,†], Gonçalo Queirós [2,†], Lesya Yefimenko [1], Filomena M. Pereira [2] and João Piedade [2,*]

[1] Instituto de Higiene e Medicina Tropical, IHMT, Universidade NOVA de Lisboa, UNL, Rua da Junqueira 100, 1349-008 Lisboa, Portugal; mariasargodinho@gmail.com (I.S.G.); a596327@gmail.com (L.Y.)

[2] Global Health and Tropical Medicine, GHTM, Associated Laboratory in Translation and Innovation Towards Global Health, LA-REAL, Instituto de Higiene e Medicina Tropical, IHMT, Universidade NOVA de Lisboa, UNL, Rua da Junqueira 100, 1349-008 Lisboa, Portugal; goncalopinhoqueiros@gmail.com (G.Q.); flmpereira@ihmt.unl.pt (F.M.P.)

* Correspondence: jp@ihmt.unl.pt

† These authors contributed equally to this work.

 https://doi.org/10.3390/microorganisms14051156

Table S1. GenBank accession numbers and subtype/recombinant form classification of each sequence analysed.

Sequence number	Subtype	Accession number	Sequence number	Subtype	Accession number
**AO7**	CRF02_AG	PX681887	**AO443**	F1/C/F1	-[1]
**AO202**	A1/H	PX681960	**AO448**	CRF124_cpx	PX681934
**AO205**	F1/J/F1	-[1]	**AO449**	CRF19_cpx/J	PX681935
**AO210**	F1/G	PX681902	**AO451**	H	PX681936
**AO235**	A8	PX681888	**AO452**	F1	PX681937
**AO237**	CRF14_BG	PX681903	**AO453**	F1/C/F1	PX681938
**AO245**	A6/A1	PX681904	**AO455**	A1	PX681939
**AO309**	CRF02_AG	PX681944	**AO456**	C/A8/C	PX681940
**AO314**	F1	PX681961	**AO462**	A2/G/A2	PX681941
**AO315**	F1	PX681962	**AO466**	CRF02_AG	PX681942
**AO318**	F1/U/F1	PX681963	**AO469**	C/L/C	-[1]
**AO319**	C/G/C	PX681964	**AO472**	F1/A7/F1	PX681894
**AO320**	C	PX681905	**AO473**	CRF02_AG	PX681895
**AO322**	F1/G	PX681906	**AO474**	D/K/D	-[1]
**AO334**	CRF18_cpx	PX681965	**AO475**	C/G/A1	PX681967
**AO356**	CRF19_cpx/G	-[1]	**AO480**	F1/G/F1	PX681896
**AO363**	CRF27_cpx	PX681907	**AO481**	F1/G/F1	PX681943
**AO364**	C/U/C	PX681908	**AO482**	CRF124_cpx	-[1]
**AO367**	D	PX681966	**AO483**	A1/G/A1	PX681945
**AO370**	C	PX681910	**AO485**	CRF20_BG	PX681893
**AO373**	CRF14_BG	PX681911	**AO488**	D	PX681909
**AO374**	A1	PX681912	**AO489**	A8	PX681946
**AO380**	F1/C	PX681913	**AO490**	C	PX681947
**AO381**	F1/A8/F1	PX681914	**AO491**	F1	PX681948
**AO382**	F1/A8/F1	PX681915	**AO492**	F1/CRF93_cpx/F1	-[1]
**AO383**	CRF11_cpx/U	PX681958	**AO493**	C	PX681897
**AO384**	CRF19_cpx/G	PX681916	**AO494**	G	PX681898
**AO386**	A2/G/A2	PX681917	**AO495**	F1/C/F1	PX681890
**AO387**	A2/G/A2	PX681918	**AO503**	C/L/C	PX681949
**AO391**	F1	PX681919	**AO507**	F1	PX681901
**AO394**	C	PX681959	**AO508**	A2	PX681950
**AO397**	C/J/C	PX681920	**AO511**	U	PX681969
**AO398**	J/CRF18_cpx/A7	PX681921	**AO512**	C	PX681951
**AO404**	A2/G/A2	PX681922	**AO515**	F1/D/F1	PX681899
**AO406**	CRF19_cpx/G	PX681923	**AO516**	C/L/C	PX681952
**AO410**	F1	PX681924	**AO519**	F1/C/F1	PX681953
**AO416**	A7	PX681925	**AO520**	G	PX681891
**AO417**	C	PX681926	**AO521**	F1/J/F1	PX681954
**AO418**	C	PX681927	**AO522**	C/L/C/L/C	-[1]
**AO423**	A8	PX681928	**AO527**	93cpx/D/A8/93cpx	PX681955
**AO432**	A8	PX681929	**AO531**	CRF02_AG	PX681970

**AO433**	U	PX681930	**AO533**	D	PX681956
**AO434**	G	PX681931	**AO536**	F1/D/F1	PX681968
**AO436**	F1/C/F1	PX681932	**AO538**	CRF124_cpx	PX681892
**AO441**	F1/CRF93_cpx/ F1	PX681933	**AO541**	F1	PX681957
**AO442**	C	PX681889	**AO545**	C	PX681900

[1] - Sequences not submitted to GenBank due to the presence of premature stop codons.

Figure S1. SimPlot bootscanning profiles of sequences submitted to recombination analysis. The red line indicates the 70% bootstrap support threshold.

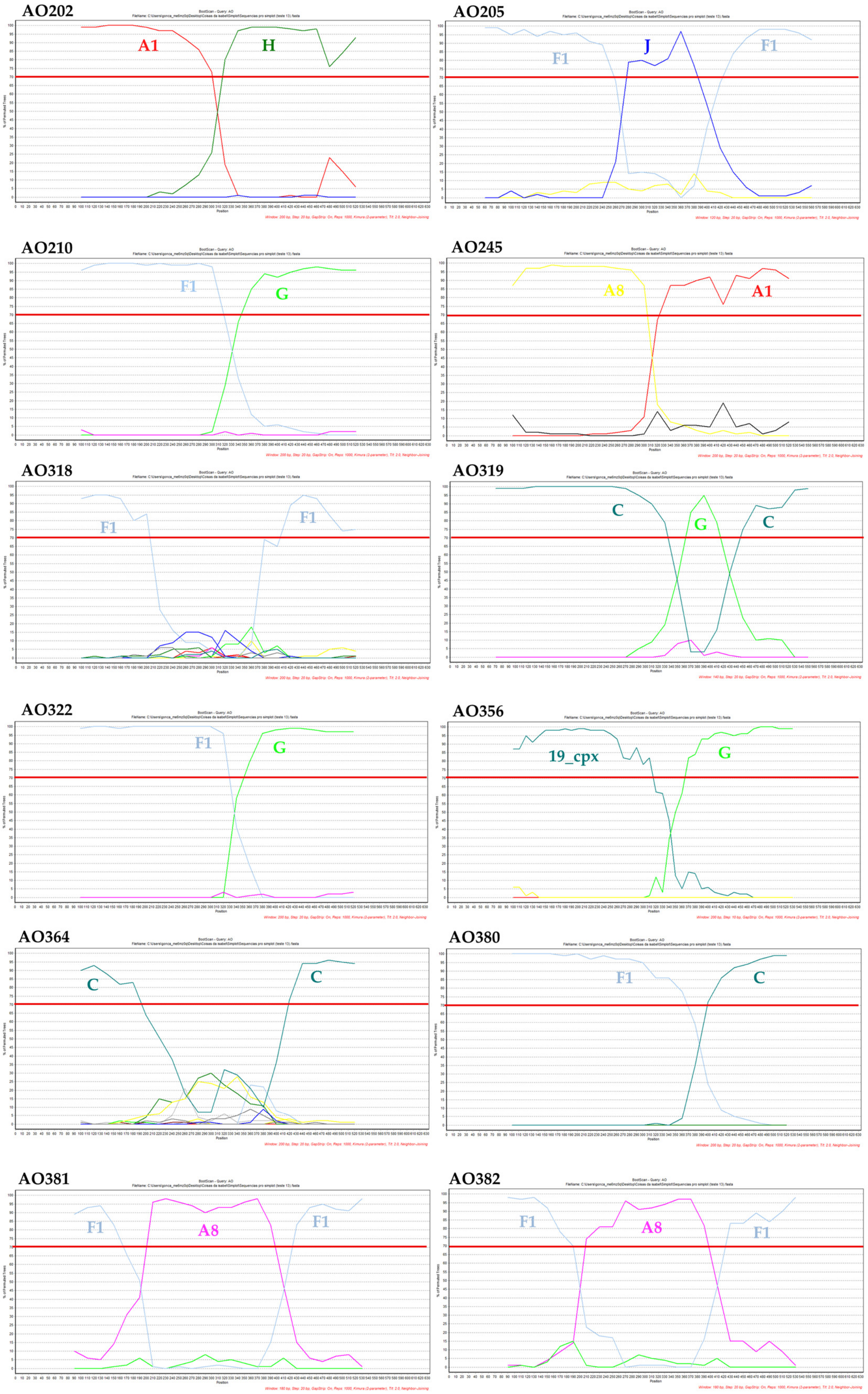

Figure S1. *Cont.*

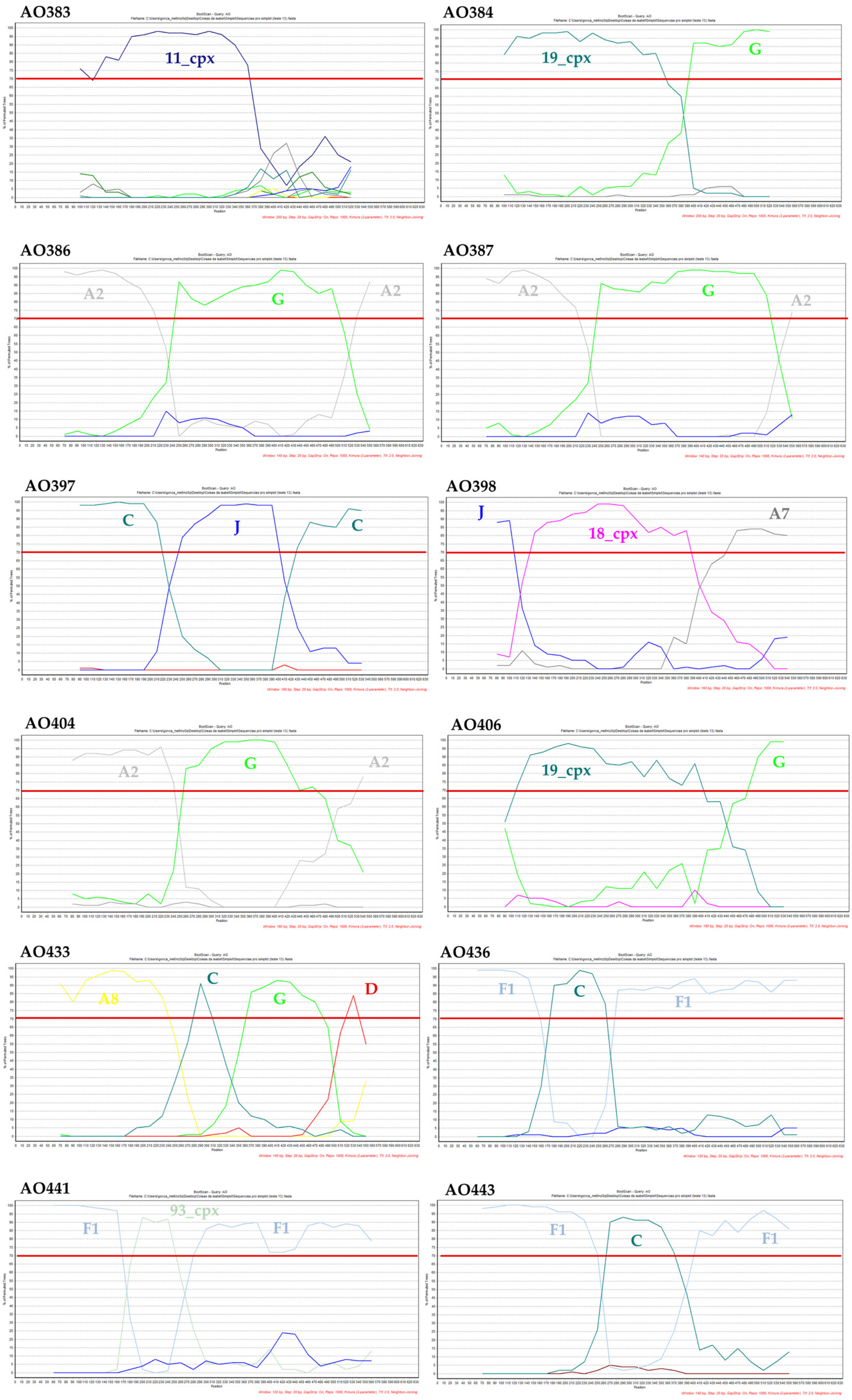

Figure S1. *Cont.*

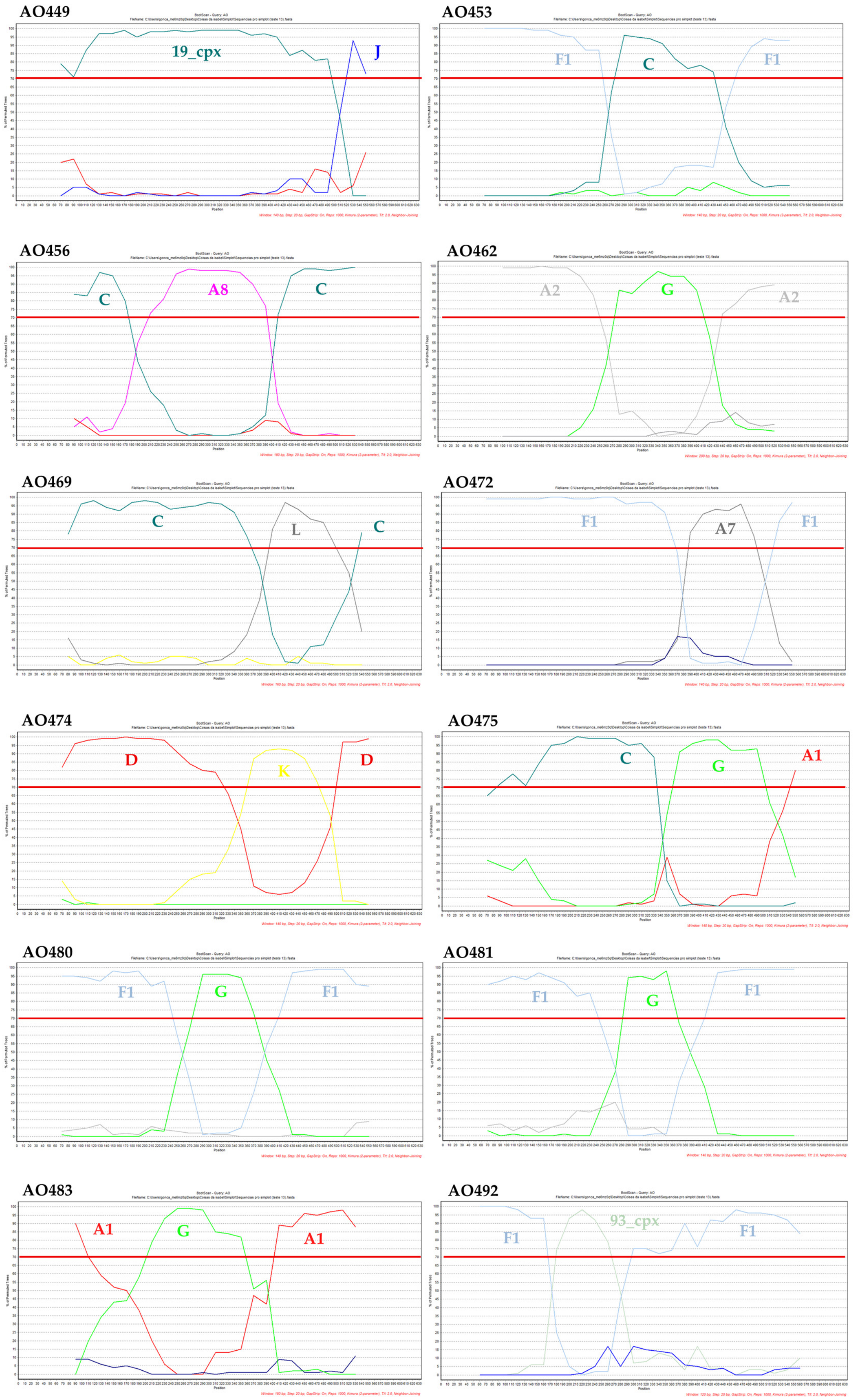

Figure S1. *Cont.*

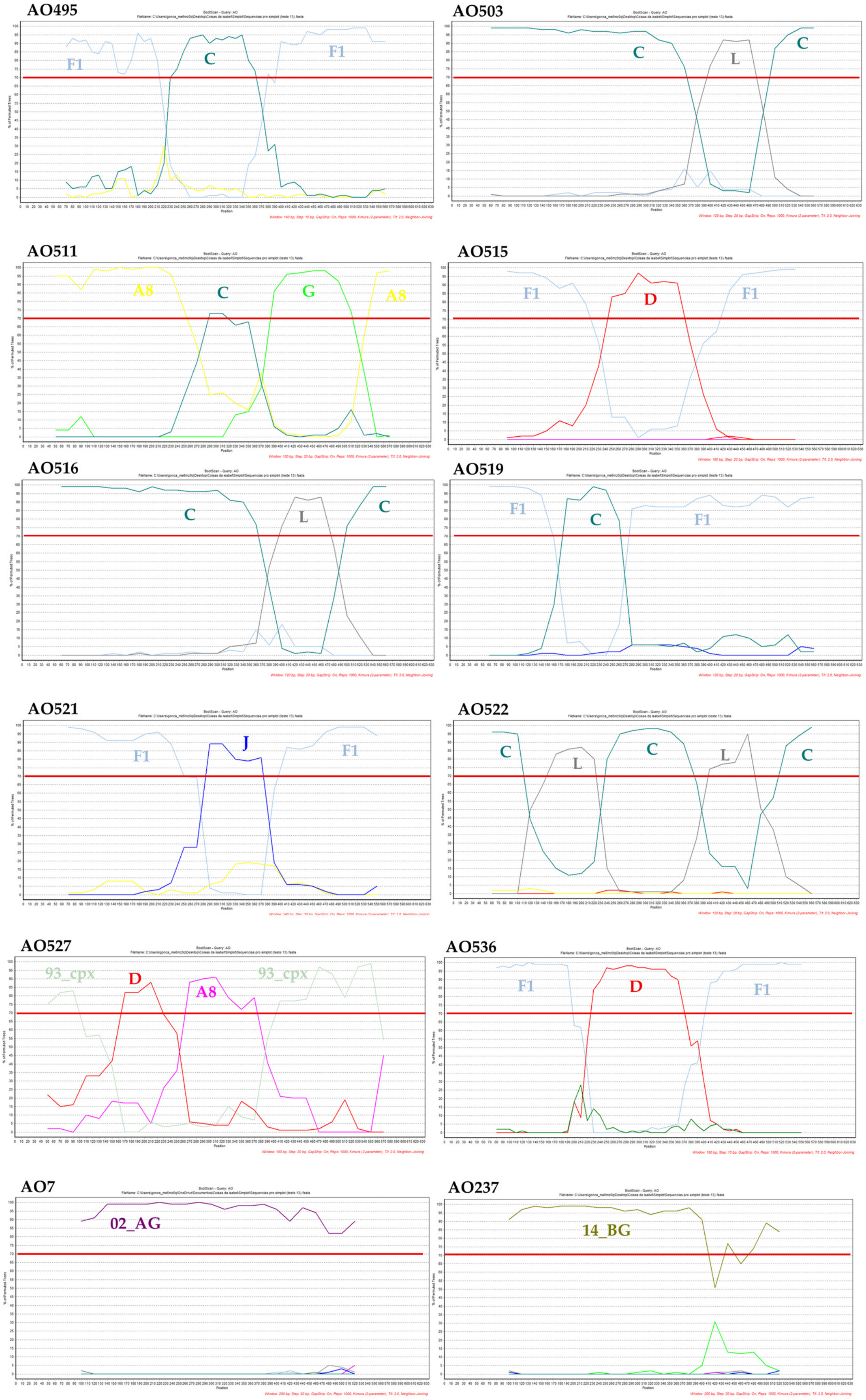

Figure S1. *Cont.*

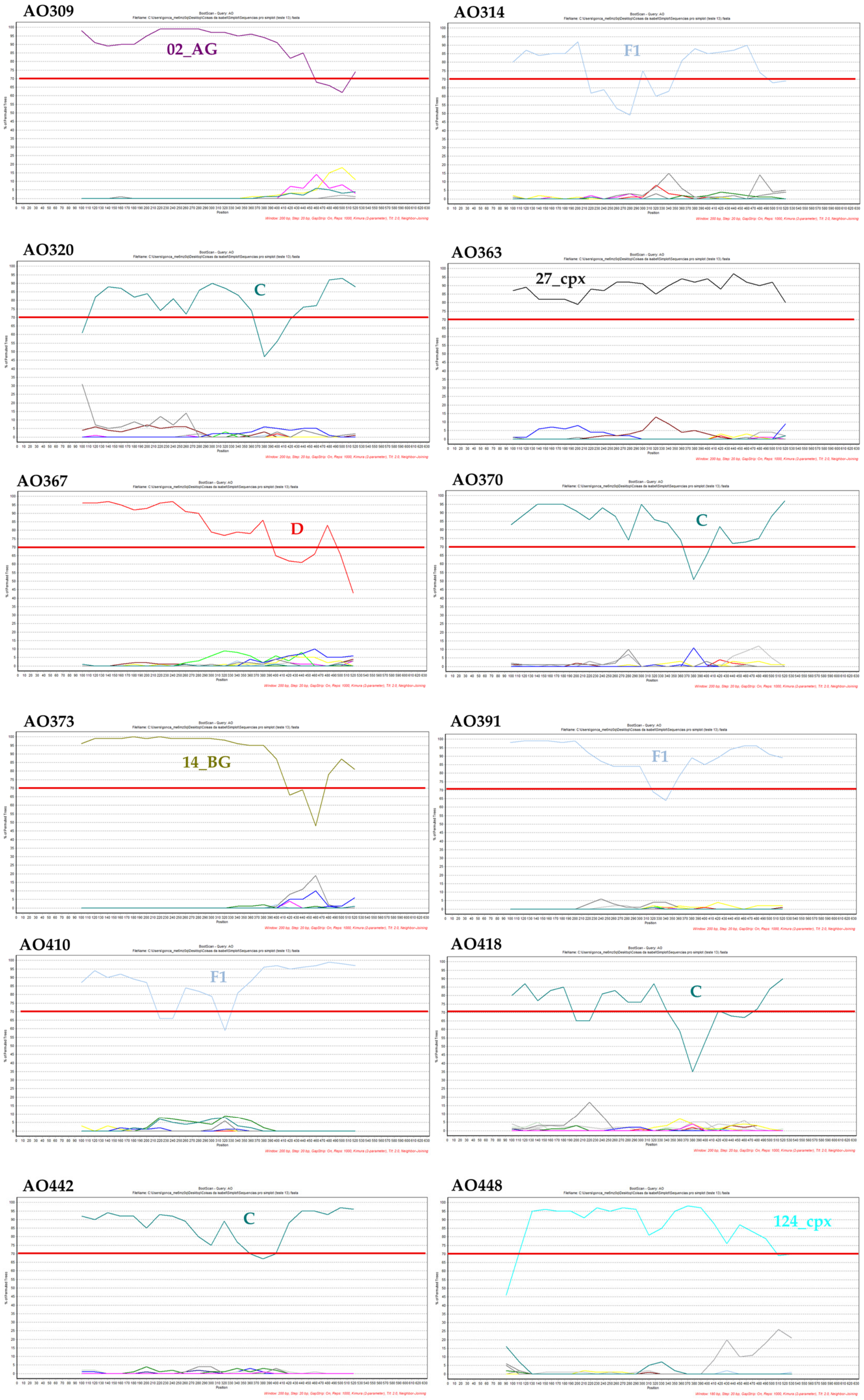

Figure S1. *Cont.*

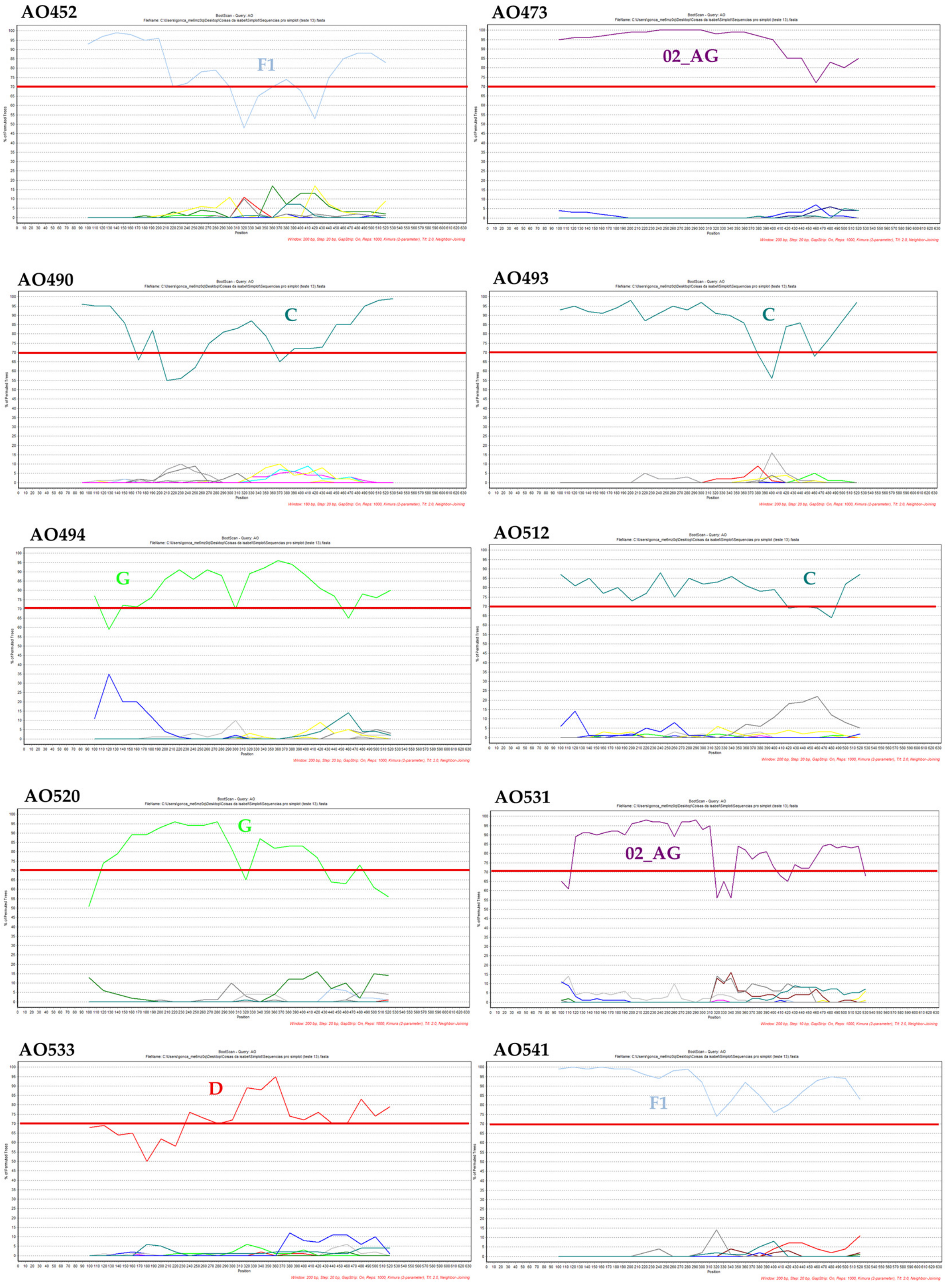

https://doi.org/10.3390/microorganisms14051156
